# Supplementary material for: Experiences and perceptions of meals on wheels volunteers in providing nutritional care to older adults: A qualitative evidence synthesis
Source: PLoS One. 2025 Apr 9;20(4):e0315443. doi: 10.1371/journal.pone.0315443 (PMC11981223; doi:10.1371/journal.pone.0315443)
Supplement: S2 Table — (DOCX) [file pone.0315443.s002.docx]

Meals on Wheels QES

Codes

| Name | Description | Files | References |
| --- | --- | --- | --- |
| Age of volunteers | This code describes the age of the volunteers as being retired, ageing themselves and struggles with physical demands of the role | 1 | 6 |
| Approach of manager to manage MoW service | This describes the approach adopted by Managers to run the MoW service | 1 | 3 |
| Being coaxed to volunteer | This code describes that some volunteers are coaxed into participation | 1 | 2 |
| Beyond food | This code describes the perception that MoW service provides more than food and nutritional benefits | 1 | 13 |
| Constant threat to MoW service | This code describes the threats to the MoW service provision that are constant and persistent. | 1 | 2 |
| COVID 19 | This code describes experiences during the pandemic. | 1 | 3 |
| Developing a bond | This code describes how a relationship is built between MoW volunteers and older adults. | 1 | 4 |
| Difficulties sourcing new volunteers | This code describes the difficulty in sourcing g new volunteers to assist with meals on wheels. | 1 | 4 |
| Driver rota | This code describes how MoW managers operate rota to accommodate the drivers in return for their time | 1 | 1 |
| Enabling older adults to live at home | This code describes the role that MoW provides to enable older adults to live in their own homes and maintain a level of independence | 1 | 8 |
| Funding | This code describes issues related to budgets and funding | 1 | 4 |
| Giving back | This code describes giving back to the community as a reason to volunteer | 1 | 5 |
| Limitations due to lack of training | This code describes the volunteers perception about training for different issues related to older adult health | 1 | 2 |
| Managers are aware of driver burden | This code describes the awareness among MoW managers of the burden drivers are under to carry out their deliveries. | 1 | 3 |
| Meeting new people | This code describes meeting new people as a reason to volunteer. | 1 | 1 |
| Multiple individual reasons to volunteer | This code describes how there are many different reasons for a person to volunteer and stay WITH MoW | 1 | 2 |
| Older adults 'shut in' at home | This code describes the reasons and vulnerability of older adults that confines them at home | 1 | 4 |
| Perceived reasons for low volunteering numbers | This code describes reasons provided by the MoW managers and staff/volunteers as to why volunteering has declined. | 1 | 1 |
| Personal benefits accrue to drivers | This code describes that personal benefits accrue to drivers and that this contributes to their staying in service. | 1 | 9 |
| Protocols skipped in effort to recruit | This code describes the deviation from usual organisation protocols that are designed to protect volunteers and service users in the effort to recruit new volunteers. | 1 | 2 |
| Providing a health and safety check for older adults | This code describes how interactions between volunteers and older adults provide a mechanism to check-in on the individuals health and safety | 1 | 12 |
| Providing a healthy meal | This code describes the provision of food and nutrition for the older adult | 1 | 1 |
| Providing nutrition care | This code describes how the MoW volunteers perform tasks to help older adults to eat the meals | 1 | 3 |
| Providing practical support to older adults | This code describes the small practical tasks that the MoW drivers might also provide to older adults in addition to delivering the meal. | 1 | 6 |
| Providing social company to older adults | This code describes how delivering the meal provides an opportunity to chat with the older adult who may be living alone | 1 | 11 |
| Relieving a burden on wider family | This code describes how a MoW service can relieve the caring burden placed on families of older adults | 1 | 1 |
| Responding to emergencies at the older adults home | This code describes how the volunteer might have to raise an alarm to bring emergency assistance to an older adult. | 1 | 2 |
| Responsibility of being a voulnteer | This code describes the feeling of responsibility that volunteers hold | 1 | 11 |
| Retention challenge faced by MoW managers | This code describes challenges faced by MoW managers in retaining volunteers. | 1 | 5 |
| Sense of community | This code describes how the MoW service is a community in and of itself. | 1 | 3 |
| Sense of satisfaction | This code describes the sense of satisfaction as a reason to volunteer. | 1 | 8 |
| Showing appreciation to drivers | This code describes how MoW managers use their limited resources to show the drivers appreciation for their time. | 1 | 4 |
| Something to do with my time | This code describes MoW volunteering as providing something to do with spare time | 1 | 2 |
| The extent to which volunteers kept the service in operation was clearly appreciated. | This code describes the critical role volunteers have to maintaining MoW service | 1 | 3 |
| Unspoken rules to remain as a volunteer | This code describes the unspoken rule of remaining as a volunteer unless you can recruit a replacement | 1 | 3 |
| Unusual effort to recruit volunteers | This code describes the methods managers resort to beyond traditional means of recruitment. Traditional means of recruitment are job advertisements or announcements asking for volunteers. | 1 | 1 |
| Volunteering is often for a considerable length of time | This code describes how long volunteers remain with MoW | 1 | 3 |
| Volunteers are sacrificing personal time | This code describes how volunteers are giving up their time for enjoying other pursuits in order to deliver meals. | 1 | 2 |
| Workload of MoW managers | This code describes the challenge of the workload maintained by MoW managers. | 1 | 2 |
