# Supplementary material for: Experiences and perceptions of meals on wheels volunteers in providing nutritional care to older adults: A qualitative evidence synthesis
Source: PLoS One. 2025 Apr 9;20(4):e0315443. doi: 10.1371/journal.pone.0315443 (PMC11981223; doi:10.1371/journal.pone.0315443)
Supplement: S3 Table — (DOCX) [file pone.0315443.s003.docx]

Full text screening outcome

| No. | Article reference | Outcome |
| --- | --- | --- |
| 1 | Yip O, Dhaini S, Esser J, Siqeca F, Mendieta MJ, Huber E, Zeller A, De Geest S, Deschodt M, Zúñiga F, Zullig LL. Health and social care of home-dwelling frail older adults in Switzerland: a mixed methods study. Bmc Geriatrics. 2022 Nov 15;22(1):857. | Exclude:  Wrong Population - Not focused on older adults; not a domiciliary older adult group |
| 2 | Waity JF. Geographic variation in barriers to the usage of food assistance in Indiana. Journal of Hunger & Environmental Nutrition. 2019 Jul 4. |  |
| 3 | Sadarangani TR, Johnson JJ, Chong SK, Brody A, Trinh-Shevrin C. Using the social ecological model to identify drivers of nutrition risk in adult day settings serving East Asian older adults. Research in gerontological nursing. 2020 May 1;13(3):146-57. |  |
| 4 | Sadarangani T, Chong S, Park S, Missaelides L, Johnson J, Trinh-Shevrin C, Brody A. A qualitative analysis of the delivery of person-centered nutrition to Asian Americans with dementia in the adult day health care setting. Journal of Applied Gerontology. 2021 Feb;40(2):179-88. |  |
| 5 | Mole L, Kent B, Hickson M, Abbott R. ‘It’s what you do that makes a difference’An interpretative phenomenological analysis of health care professionals and home care workers experiences of nutritional care for people living with dementia at home. BMC geriatrics. 2019 Dec;19:1-0. |  |
| 6 | Mole L, Kent B, Abbott R, Hickson M. Family carers’ experiences of nutritional care for people living with dementia at home: An interpretative phenomenological analysis. Dementia. 2021 Jan;20(1):231-46. |  |
| 7 | Mawardi F, Lestari AS, Kusnanto H, Sasongko EP, Hilmanto D. Malnutrition in older adults: how interprofessional teams see it? A systematic review of the qualitative research. Family Practice. 2021 Feb 1;38(1):43-8. |  |
| 8 | Mahony LO, Shea EO, O'Connor EM, Tierney A, Harkin M, Harrington J, Kennelly S, Arendt E, O'Toole PW, Timmons S. ‘Good, honest food’: older adults' and healthcare professionals' perspectives of dietary influences and food preferences in older age in Ireland. Journal of Human Nutrition and Dietetics. 2023 Oct;36(5):1833-44. |  |
| 9 | Lee JS, Frongillo EA, Olson CM. Understanding targeting from the perspective of program providers in the elderly nutrition program. Journal of Nutrition for the Elderly. 2005 Apr 14;24(3):25-45. |  |
| 10 | Demons JL, Sink KM, Applegate WB, Davis BR. Early Outcomes of a Novel Senior Mentor Program Utilizing Meals on Wheels Volunteers. In Journal of the American Geriatrics Society 2011 Apr 1 (Vol. 59, Pp. S85-S85). Commerce Place, 350 Main St, Malden 02148, Ma Usa: Wiley-Blackwell. |  |
| 11 | Dave JM, Thompson DI, Svendsen-Sanchez A, McNeill LH, Jibaja-Weiss M. Development of a nutrition education intervention for food bank clients. Health Promotion Practice. 2017 Mar;18(2):221-8. |  |
| 12 | Canales MK, Coffey N, Moore E. Exploring health implications of disparities associated with food insecurity among low-income populations. Nursing Clinics. 2015 Sep 1;50(3):465-81. |  |
| 13 | Bjørner T, Korsgaard D, Reinbach HC, Perez-Cueto FJ. A contextual identification of home-living older adults' positive mealtime practices: A honeycomb model as a framework for joyful aging and the importance of social factors. Appetite. 2018 Oct 1;129:125-34. |  |
| 14 | Agrawal S, Makuch S, Dróżdż M, Strzelec B, Sobieszczańska M, Mazur G. The impact of the COVID-19 emergency on life activities and delivery of healthcare services in the elderly population. Journal of Clinical Medicine. 2021 Sep 10;10(18):4089. |  |
| 15 | Aburrow A, Wallis K, Steward K, Cholet A, Murphy JL. Managing malnutrition (as undernutrition) and caring for older people living in the community: The development and publishing of a new workbook and training videos for staff working in community teams (eg nursing, integrated and therapy teams). Clinical Nutrition ESPEN. 2020 Feb 1;35:247-8. |  |
| 16 | Wingrove K, Barbour L, Palermo C. Exploring nutrition capacity in Australia's charitable food sector. Nutrition & Dietetics. 2017 Nov;74(5):495-501. | Exclude:  Wrong outcome - Not the samples perspective |
| 17 | Westcott JB, Fullen MC, Tomlin CC, Eikenberg K, Delaughter PM, Mize MC, Shannonhouse LR. ‘Listen closer’: home-delivered meal volunteers’ understanding of their role in suicide intervention. Ageing & Society. 2024 Mar;44(3):642-60. |  |
| 18 | Warburton J, Moore M, Oppenheimer M. Challenges to the recruitment and retention of volunteers in traditional nonprofit organizations: a case study of Australian meals on wheels. International Journal of Public Administration. 2018 Dec 10;41(16):1361-73. |  |
| 19 | Van Dijk HM, Cramm JM, Nieboer AP. The experiences of neighbour, volunteer and professional support‐givers in supporting community dwelling older people. Health & social care in the community. 2013 Mar;21(2):150-8. |  |
| 20 | Ulsperger JS, McElroy J, Robertson H, Ulsperger K. Senior companion program volunteers: Exploring experiences, transformative rituals, and recruitment/retention issues. Qualitative Report. 2015;20(9):1458. |  |
| 21 | Morris AM, Engelberg JK, Schmitthenner B, Dosa D, Gadbois E, Shield RR, Akobundu U, Thomas KS. Leveraging home‐delivered meal programs to address unmet needs for at‐risk older adults: preliminary data. Journal of the American Geriatrics Society. 2019 Sep;67(9):1946-52. |  |
| 22 | Pardasani M. Motivation to volunteer among senior center participants. Journal of gerontological social work. 2018 Apr 3;61(3):313-33. |  |
| 23 | Brady PJ, Askelson NM, Thompson H, Kersten S, Hopkins H. Meeting older adults’ food needs: Interviews with area agency on aging staff, food bank staff, and older adults. Journal of nutrition in gerontology and geriatrics. 2022 Jul 3;41(3):235-55. |  |

| 24 | Choi NG, Lee A, Goldstein M. Meals on Wheels: Exploring potential for and barriers to integrating depression intervention for homebound older adults. Home health care services quarterly. 2011 Oct 1;30(4):214-30. | Exclude:  Wrong study design - Not qualitative or able to extract qual data separately; not an empirical study/primary research; a systematic review; not meal delivery to home; not nutrition related |
| --- | --- | --- |
| 25 | Latif J, Dabbous M, Weekes CE, Baldwin C. The effectiveness of trained volunteer delivered interventions in adults at risk of malnutrition: a systematic review and meta-analysis. Clinical Nutrition. 2021 Mar 1;40(3):710-27. |  |
| 26 | Lee JS, Frongillo EA, Keating MA, Deutsch LH, Daitchman J, Frongillo DE. Targeting of home-delivered meals programs to older adults in the United States. Journal of Nutrition for the Elderly. 2008 Sep 16;27(3-4):405-15. |  |
| 27 | Mole L, Kent B, Abbott R, Wood C, Hickson M. The nutritional care of people living with dementia at home: A scoping review. Health & social care in the community. 2018 Jul;26(4):e485-96. |  |
| 28 | Mousa TY, Freeland-Graves JH. Motivations for volunteers in food rescue nutrition. Public health. 2017 Aug 1;149:113-9. |  |
| 29 | Northridge ME, Kum SS, Chakraborty B, Greenblatt AP, Marshall SE, Wang H, Kunzel C, Metcalf SS. Third places for health promotion with older adults: using the consolidated framework for implementation research to enhance program implementation and evaluation. Journal of Urban Health. 2016 Oct;93:851-70. |  |
| 30 | Lo DT, Larson C, Kaminishi K, Rogers SE, Tong AK, Hendre A, Williams B, Rivera J. Friends of the Elderly: Geriatric Education for Community Volunteers. In Journal Of The American Geriatrics Society 2015 Apr 1 (Vol. 63, pp. S46-S46). 111 River St, Hoboken 07030-5774, Nj Usa: Wiley-Blackwell. | Exclude:  Wrong publication type - Not peer reviewed article; conference abstract, no full text available |
| 31 | Williams, L., Building community capacity to support healthy eating: An evaluation of the nutrition skills for lifetm nutrition training programme in wales. Revista Espanola de Nutricion Humana y Dietetica, 2016. 20: p. 377-378 |  |
| 32 | Walton, K., et al., Bridging the gap between hospital and home: Exploring and developing a 'Pantry Box' for meals on wheels clients. Revista Espanola de Nutricion Humana y Dietetica, 2016. 20: p. 556 |  |
| 33 | Reinert, R., et al., Malnutrition in the elderly: Attitudes of employees of the Geneva institution homecare (IMAD). Clinical Nutrition, 2014. 33: p. S229. |  |
| 34 | Murphy, J.L., et al., Using the Nutrition Wheel to identity risk of malnutrition among older adults in the community: a process evaluation. Clinical Nutrition ESPEN, 2022. 48: p. 487-488 |  |
| 35 | O'Dwyer, C. and V. Timonen, Doomed to Extinction? The Nature and Future of Volunteering for Meals-on-Wheels Services. Voluntas, 2009. 20(1): p. 35-49. | Include |
| 36 | Thomas, K.S., et al., “It’s Not Just a Simple Meal. It’s So Much More”: Interactions Between Meals on Wheels Clients and Drivers. Journal of Applied Gerontology, 2020. 39(2): p. 151-158. |  |
| 37 | Papadaki, A., et al., ‘It’s not just about the dinner; it’s about everything else that we do’: A qualitative study exploring how Meals on Wheels meet the needs of self-isolating adults during COVID-19. Health and Social Care in the Community, 2022. 30(5): p. e2012-e2021 |  |
