# Supplementary material for: Experiences and perceptions of meals on wheels volunteers in providing nutritional care to older adults: A qualitative evidence synthesis
Source: PLoS One. 2025 Apr 9;20(4):e0315443. doi: 10.1371/journal.pone.0315443 (PMC11981223; doi:10.1371/journal.pone.0315443)
Supplement: S4 Table — (DOCX) [file pone.0315443.s004.docx]

The experiences and perceptions of community volunteers in providing nutrition care supporting older adults. Qualitative evidence synthesis

SPIDER Framework for search strategy of the research question (Cook et al 2012):

| **Sample** | Those persons who deliver meals to older adults at home/in community. They might offer this service voluntarily, on retention, or salaried or a combination. The meal delivery service may be part of a healthcare system (e.g. healthcare assistance), community scheme (e.g. meals on wheels), or charitable donations (e.g. food hampers). These services could be implemented from public or private facilities.  Given the reviews focus, i.e. the community volunteer who deliver meals, we will exclude the perceptions and experience of clients of these services. |
| --- | --- |
| **Phenomenon of Interest** (to understand the how and why of individual experiences) | Provision of nutrition care, including the awareness, identification and intervention measures reported. This might include observation by food delivery persons of nutrition-related contributary factors (e.g. transportation, access, availability, skills), signs (e.g. poor appetite, swallow difficulty), symptoms (e.g. weight loss, loose fitting jewellery, pallor, health complaints), finance (e.g. contributions, pension, allowances, etc.) among older adults living in community.  The settings of these phenomena are the domiciliary home (individual level) or daycare setting in an older person local to their homes (community level). |
| **Design** (to help make decisions about the robustness of the study and analysis) | Interview, focus groups, document analysis and observations |
| **Evaluation** (outcomes are subjective- attitudes, views, etc.) | Experience and Perceptions of nutrition care and service provider/volunteers’ individual role in care pathways that support older adults to remain at home. |
| **Research type** | Qualitative, mixed method (where qualitative data analysis can be extracted) including phenomenology, ethnography, grounded theory, case studies, etc. |

Cooke A, Smith D, Booth A. Beyond PICO: The SPIDER Tool for Qualitative Evidence Synthesis. Qualitative Health Research. 2012;22(10):1435-1443. doi:10.1177/1049732312452938

Seven databases: Scopus, PubMed, CINAHL, Web of Science, Embase, MEDLINE, and PsycINFO

Search terms:

| Original search terms developed August 2022 | | | |
| --- | --- | --- | --- |
| Community voluntary providers | Nutrition Care | Older adults | Qualitative |
| MeSH | | | |
| MM Volunteer Workers  MM Volunteer Experiences  MM Communities  MM Community Networks  MM Home Health Aides | MM Food Assistance  MM Meals-on-Wheels (Saba CCC)  MM Meal Preparation  MM Meals | MM Frail Elderly  MM Aged | MM Qualitative Studies |
| Free text search | | | |
| “Community volunteers” OR community volunt* OR volunteers OR “voluntary provider” OR “community voluntary provider” OR  “Non-governmental organization” OR “NGO” | “Malnutrition” OR “malnourished” OR maln* OR “undernutrition” OR “poor nutrition status” OR “nutrition care” OR “nutrition care pathway” OR “identify malnutrition” OR “detect malnutrition” OR “meal provision” OR “meal preparation” OR grocery OR “grocery shopping” OR “grocery provision” OR “food shopping” | “Elderly” OR “older adult” OR senior* OR geriatric* OR “aged individual” OR “aged people” OR “aging” OR “older people” OR “older individual” OR “over 65” OR “sixty five and over” | Qualitative OR “mixed methods” OR “observational study” OR “survey” OR “case studies” OR “evaluation methods” OR interview* OR “focus group*” OR “naturalistic observation” OR “participant observation” OR “social science research” OR transcript* OR ethnography* OR phenomenol* OR “grounded theory*” OR “purposive sample” OR hermeneutic* OR heuristic* OR “lived experience*” OR narrative*  OR “life experience*” OR “life stor*” OR “cluster sample” OR “action research” OR “observational method” OR “content analysis” OR “thematic  analysis” OR “narrative analysis” OR “constant comparative method” OR “field stud*” OR “fieldnotes” OR “audio recording” OR “video recording” OR “theoretical sample” OR “discourse analysis” |
| Additional suggested search terms based on update August 2023 | | | |
| “community health” OR “community care” OR “domiciliary care” OR “domiciliary support” | “meals on wheels” OR “home-delivered meals” OR “community meals” | “ageing” OR “homebound older” OR “house bound older” OR “frailty” |  |

*Eligibility*

Inclusion criteria:

- Primary research and qualitative studies (or mixed-methods studies if qualitative results were reported separately) where volunteers’ perceptions and experiences while providing food and meal support to community dwelling older adults (65 years and older) is described.
- Studies are peer-reviewed, published articles in English and Spanish languages relating to the nutritional impact, such as but not limited to recognition of issues with eating, drinking or appetite, of food and meal provision services delivered by volunteers.
- Volunteers/service providers may be described as paid or voluntary or a combination of both.
- Provision of food at day care centers that older adults access voluntarily.

Exclusion

- Studies were conducted in developing countries.
- Studies that describe the experiences of service providers who carry out provision of medicines, prescriptions, medical dressings or treatments.
- Studies that describe meal provision at a central venue (meals are not delivered to the individuals home), such as respite/rehabilitation centres.
- Where meals are not delivered in-person, e.g. postal system used.

Included articles (original FYP):

O’Dwyer, C. and Timonen, V. (2009) ‘Doomed to extinction? The nature and future of volunteering for meals-on-wheels services’, *VOLUNTAS: International Journal of Voluntary and Nonprofit Organizations*, 20, 35-49, available: <http://dx.doi.org/10.1007/s11266-008-9074-8>.

Thomas, K.S., Gadbois, E.A., Shield, R.R., Akobundu, U., Morris, A.M. and Dosa, D.M. (2020) ‘“It’s not just a simple meal. It’s so much more”: Interactions between meals on wheels clients and drivers’, *Journal of Applied Gerontology*, 39(2), 151-158, available: <http://dx.doi.org/10.1177/0733464818820226>.

Appendix 1 (initial searches update):

CINAHL Complete (Ebsco) completed 17/08/23

| S1 | (MM "Volunteer Workers/HI/EV/EI/ED/LS/OG/PF/ST/TD/UT/CL") OR (MM "Volunteer Experiences/CL/ED/EP/EI/EH/EV/HI/TD") OR (MM "Communities/CL/ED/EI/EV/HI/PF/ST/TD/UT/MT") OR (MM "Community Networks/CL/ED/EI/EV/HI/LS/OG/PF/ST/TD/UT") OR (MM "Home Health Aides/CL/EI/ED/EV/HI/OG/PF") | 2,471 |
| --- | --- | --- |
| S2 | TI ( “Community volunteers” OR "community volunt*" OR volunt* OR “voluntary provider” OR “community voluntary provider” OR “Non-governmental organi#ation” OR “NGO” ) OR AB ( “Community volunteers” OR "community volunt*" OR volunt* OR “voluntary provider” OR “community voluntary provider” OR “Non-governmental organi#ation” OR “NGO” ) | 76,338 |
| S3 | TI ( “community health” OR “community care” OR “domiciliary care” OR “domiciliary support” ) OR AB ( “community health” OR “community care” OR “domiciliary care” OR “domiciliary support” ) | 22,730 |
| S4 | S1 OR S2 OR S3 | 99,657 |
| S5 | (MM "Food Assistance/EC/ED/EP/EI/EV/HI/LS/OG/PF/ST/TD/UT/CL") OR (MM "Meals-on-Wheels (Saba CCC)/ED/EV/UT") OR (MM "Meal Preparation/EC/ED/EI/EV/LS/OG/PF/ST/TD/UT") OR (MM "Meals/CL/EC/ED/EI/ST/UT") | 362 |
| S6 | TX “Malnutrition” OR “malnourished” OR maln* OR “undernutrition” OR “poor nutrition status” OR “nutrition care” OR “nutrition care pathway” OR “identify malnutrition” OR “detect malnutrition” OR “meal provision” OR “meal preparation” OR grocery OR “grocery shopping” OR “grocery provision” OR “food shopping” | 66,608 |
| S7 | TX “meals on wheels” OR “home-delivered meals” OR “community meals” | 1668 |
| S8 | S5 OR S6 OR S7 | 67,969 |
| S9 | (MM "Frail Elderly/CL/ED/EI/PF") OR (MM "Aged/CL/ED/EI/EV/PF") | 1,215 |
| S10 | TI ( “Elderly” OR “older adult” OR senior* OR geriatric* OR “aged individual” OR “aged people” OR “aging” OR “older people” OR “older individual” OR “over 65” OR “sixty five and over” ) OR AB ( “Elderly” OR “older adult” OR senior* OR geriatric* OR “aged individual” OR “aged people” OR “aging” OR “older people” OR “older individual” OR “over 65” OR “sixty five and over” ) | 234,727 |
| S11 | TI ( “ageing” OR “homebound older” OR “house bound older” OR “frailty” ) OR AB ( “ageing” OR “homebound older” OR “house bound older” OR “frailty” ) | 29, 686 |
| S12 | S9 OR S10 OR S11 | 253,455 |
| S13 | (MM "Qualitative Studies") | 3,700 |
| S14 | TI ( Qualitative OR “mixed methods” OR “observational study” OR “survey” OR “case studies” OR “evaluation methods” OR interview* OR “focus group*” OR “naturalistic observation” OR “participant observation” OR “social science research” OR transcript* OR ethnography* OR phenomenol* OR “grounded theory*” OR “purposive sample” OR hermeneutic* OR heuristic* OR “lived experience*” OR narrative* OR “life experience*” OR “life stor*” OR “cluster sample” OR “action research” OR “observational method” OR ... | 782,923 |
| S15 | S13 OR S14 | 783,415 |
| S16 | S4 AND S8 AND S12 AND S15 | 76 |

Medline (OVID) search completed 18/08/2023

| S1 | Volunteer worker.mp. or exp Volunteers/ | 38,035 |
| --- | --- | --- |
| S2 | residence characteristics/ or home environment/ or independent living/ | 49,542 |
| S3 | (communities or #2).mp. [mp=title, book title, abstract, original title, name of substance word, subject heading word, floating sub-heading word, keyword heading word, organism supplementary concept word, protocol supplementary concept word, rare disease supplementary concept word, unique identifier, synonyms, population supplementary concept word, anatomy supplementary concept word] | 688,294 |
| S4 | Community Networks/cl, ec, es, hi, og, st, td [Classification, Economics, Ethics, History, Organization & Administration, Standards, Trends] | 3490 |
| S5 | Home Health Aides/cl, ec, ed, es, og, px, st, td [Classification, Economics, Education, Ethics, Organization & Administration, Psychology, Standards, Trends] | 452 |
| S6 | (Community volunteers or community volunt* or volunteers or voluntary provider or community voluntary provider or Non-governmental organi#ation or NGO).ab,ti. | 189,873 |
| S7 | (community health or community care or domiciliary care or domiciliary support).ab,ti. | 35,320 |
| S8 | 1 or 2 or 3 or 4 or 5 or 6 or 7 | 7,066,108 |
| S9 | exp Food Assistance/cl, ec, es, hi, og, st, td [Classification, Economics, Ethics, History, Organization & Administration, Standards, Trends] | 403 |
| S10 | meals on wheels.mp. or Food Services/ | 6,278 |
| S11 | exp Meals/ or Meal preparation.mp. | 9,217 |
| S12 | (Malnutrition or malnourished or maln* or undernutrition or poor nutrition status or nutrition care or nutrition care pathway or identify malnutrition or detect malnutrition or meal provision or meal preparation or grocery or grocery shopping or grocery provision or food shopping).ab,ti. | 66,026 |
| S13 | (meals on wheels or home-delivered meals or community meals).ab,ti. | 351 |
| S14 | 9 or 10 or 11 or 12 or 13 | 80,092 |
|  | exp Aged/ | 3,459,049 |
|  | ((Elderly or older adult or senior* or geriatric* or aged individual or aged people or aging or older people or older individual or over 65 or sixty five) and over).tw. | 91,152 |
|  | (ageing or homebound older or house bound older or frailty).tw. | 74,766 |
|  | 15 or 16 or 17 | 3,535,340 |
|  | exp Qualitative Research/ | 82,892 |
|  | (Qualitative or mixed methods or observational study or survey or case studies or evaluation methods or interview* or focus group* or naturalistic observation or participant observation or social science research or transcript* or ethnography* or phenomenol* or grounded theory* or purposive sample or hermeneutic* or heuristic* or lived experience* or narrative* or life experience* or life stor* or cluster sample or action research or observational method or content analysis or thematic analysis or narrative analysis or constant comparative method or field stud* or fieldnotes or audio recording or video recording or theoretical sample or discourse analysis).ab,ti. | 2,596,151 |
|  | 19 or 20 | 2,601,393 |
|  | 8 and 14 and 18 and 21 | 1088 |
|  |  |  |

Embase search completed 18/08/2023

| S1 | (**'voluntary worker'**/exp OR **'voluntary worker'**) AND **'voluntary worker'**/exp | 5,709 |
| --- | --- | --- |
| S2 | **'community'**/exp OR **'community care'**/exp | 226,179 |
| S3 | 'home health agency'/exp OR 'home care'/exp | 90,284 |
| S4 | ('community volunteers' OR community) AND volunt* OR volunteers OR 'voluntary provider' OR 'community voluntary provider' OR 'non-governmental organization' OR 'ngo' OR 'volunteer experiences' OR 'home health aides' OR 'community networks':ab,ti | 288,828 |
| S5 | 'community health' OR 'community care' OR 'domiciliary care' OR 'domiciliary support':ab,ti | 243,850 |
| S6 | #1 OR #2 OR #3 OR #4 OR #5 | 732,696 |
| S7 | 'food assistance'/exp | 2,523 |
| S8 | 'home delivered meal'/exp | 127 |
| S9 | 'meal'/de | 22,351 |
| S10 | 'malnutrition' OR 'malnourished' OR maln* OR 'undernutrition' OR 'poor nutrition status' OR 'nutrition care' OR 'nutrition care pathway' OR 'identify malnutrition' OR 'detect malnutrition' OR 'meal provision' OR 'meal preparation' OR grocery OR 'grocery shopping' OR 'grocery provision' OR 'food shopping':kw | 123,761 |
| S11 | 'meals on wheels' OR 'home-delivered meals' OR 'community meals':kw | 507 |
| S12 | #7 OR #8 OR #9 OR #10 OR #11 | 147,666 |
| S13 | 'aged'/exp | 3,756,971 |
| S14 | 'frail elderly'/exp | 12,226 |
| S15 | 'elderly' OR 'older adult' OR senior* OR geriatric* OR 'aged individual' OR 'aged people' OR 'aging' OR 'older people' OR 'older individual' OR 'over 65' OR 'sixty five and over':kw | 1,797,111 |
| S16 | 'ageing' OR 'homebound older' OR 'house bound older' OR 'frailty':kw | 140,278 |
| S17 | #13 OR #14 OR #15 OR #16 | 4,750,218 |
| S18 | 'qualitative research'/exp | 117,645 |
| S19 | qualitative:ab,ti OR 'mixed methods':ab,ti OR 'observational study':ab,ti OR 'survey':ti,ab OR 'case studies':ab,ti OR 'evaluation methods':ti,ab OR interview*:ab,ti OR 'focus group*':ti,ab OR 'naturalistic observation':ab,ti OR 'participant observation':ab,ti OR 'social science research':ab,ti OR transcript*:ab,ti OR ethnography*:ab,ti OR phenomenol*:ab,ti OR 'grounded theory*':ab,ti OR 'purposive sample':ab,ti OR hermeneutic*:ab,ti OR heuristic*:ab,ti OR 'lived experience*':ab,ti OR narrative*:ab,ti OR 'life experience*':ab,ti OR 'life stor*':ab,ti OR 'cluster sample':ab,ti OR 'action research':ab,ti OR 'observational method':ab,ti OR 'content analysis':ab,ti OR 'thematic analysis':ab,ti OR 'narrative analysis':ab,ti OR 'constant comparative method':ab,ti OR 'field stud*':ab,ti OR 'fieldnotes':ab,ti OR 'audio recording':ab,ti OR 'video recording':ab,ti OR 'theoretical sample':ab,ti OR 'discourse analysis':ab,ti | 3,261,092 |
| S20 | #18 OR #19 | 3,269,608 |
| S21 | #6 AND #12 AND #17 AND #20 | 615 |
|  |  |  |
|  |  |  |

Web of Science search 18082023

| S1 | volunteer workers (Topic) or volunteer experiences (Topic) or Communities (Topic) or Community network* (Topic) or Home Health Aide* (Topic) | 1,635,319 |
| --- | --- | --- |
| S2 | (TI=(“Community volunteers” OR community volunt* OR volunteers OR “voluntary provider” OR “community voluntary provider” OR “Non-governmental organi#ation” OR “NGO” )) AND AB=(“Community volunteers” OR community volunt* OR volunteers OR “voluntary provider” OR “community voluntary provider” OR “Non-governmental organization” OR “NGO” ) | 18,986 |
|  | (TI=(“community health” OR “community care” OR “domiciliary care” OR “domiciliary support”)) OR AB=(“community health” OR “community care” OR “domiciliary care” OR “domiciliary support”) | 32,044 |
|  | #3 OR #2 OR #1 | 1,649,881 |
|  | TS=("Food assistance" OR "meals on wheels" OR "Meal preparation" OR "Meals") | 121,132 |
|  | ALL=(“meals on wheels” OR “home-delivered meals” OR “community meals”) | 380 |
|  | ALL=(Free text search “Malnutrition” OR “malnourished” OR maln* OR “undernutrition” OR “poor nutrition status” OR “nutrition care” OR “nutrition care pathway” OR “identify malnutrition” OR “detect malnutrition” OR “meal provision” OR “meal preparation” OR grocery OR “grocery shopping” OR “grocery provision” OR “food shopping” ) | 85,635 |
|  | #5 OR #6 OR #7 | 203,677 |
|  | (TS=(Frail elderly)) OR TS=(aged) | 4,038,463 |
|  | (TI=(“Elderly” OR “older adult” OR senior* OR geriatric* OR “aged individual” OR “aged people” OR “aging” OR “older people” OR “older individual” OR “over 65” OR “sixty five and over” )) OR AB=(“Elderly” OR “older adult” OR senior* OR geriatric* OR “aged individual” OR “aged people” OR “aging” OR “older people” OR “older individual” OR “over 65” OR “sixty five and over” ) | 4,189,386 |
|  | (TI=(“ageing” OR “homebound older” OR “house bound older” OR “frailty”)) OR AB=(“ageing” OR “homebound older” OR “house bound older” OR “frailty”) | 3,867,395 |
|  | #9 OR #10 OR #11 | 4,361,529 |
|  | TS=(Qualitative research) | 241,994 |
|  | (TI=(Qualitative OR “mixed methods” OR “observational study” OR “survey” OR “case studies” OR “evaluation methods” OR interview* OR “focus group*” OR “naturalistic observation” OR “participant observation” OR “social science research” OR transcript* OR ethnography* OR phenomenol* OR “grounded theory*” OR “purposive sample” OR hermeneutic* OR heuristic* OR “lived experience*” OR narrative* OR “life experience*” OR “life stor*” OR “cluster sample” OR “action research” OR “observational method” OR “content analysis” OR “thematic analysis” OR “narrative analysis” OR “constant comparative method” OR “field stud*” OR “fieldnotes” OR “audio recording” OR “video recording” OR “theoretical sample” OR “discourse analysis”)) OR AB=(Qualitative OR “mixed methods” OR “observational study” OR “survey” OR “case studies” OR “evaluation methods” OR interview* OR “focus group*” OR “naturalistic observation” OR “participant observation” OR “social science research” OR transcript* OR ethnography* OR phenomenol* OR “grounded theory*” OR “purposive sample” OR hermeneutic* OR heuristic* OR “lived experience*” OR narrative* OR “life experience*” OR “life stor*” OR “cluster sample” OR “action research” OR “observational method” OR “content analysis” OR “thematic analysis” OR “narrative analysis” OR “constant comparative method” OR “field stud*” OR “fieldnotes” OR “audio recording” OR “video recording” OR “theoretical sample” OR “discourse analysis”) | 5,106,929 |
|  | #13 OR #14 | 5,111,812 |
|  | #15 AND #12 AND #8 AND #4 | 2,132 |

APA Psych INFO (Ebsco) search completed 18082023

|  | MM "Volunteers" OR DE "Assistance (Social Behavior)" OR DE "Bystander Effect" OR DE "Social Support" OR DE "Volunteers" OR DE "Community Involvement" OR DE "Collective Efficacy Theory" | 84,885 |
| --- | --- | --- |
|  | MM "Community Services" OR DE "Home Care" OR DE "Home Visiting Programs" | 24,034 |
|  | MM "Paraprofessional Personnel" OR DE "Caregivers" | 44,298 |
|  | TI ( “Community volunteers” OR community volunt* OR volunteers OR “voluntary provider” OR “community voluntary provider” OR “Non-governmental organi#ation” OR “NGO” ) OR AB ( “Community volunteers” OR community volunt* OR volunteers OR “voluntary provider” OR “community voluntary provider” OR “Non-governmental organi#ation” OR “NGO” ) | 39,782 |
|  | TI ( “community health” OR “community care” OR “domiciliary care” OR “domiciliary support” ) OR AB ( “community health” OR “community care” OR “domiciliary care” OR “domiciliary support” ) | 10,801 |
|  | S1 OR S2 OR S3 OR S4 OR S5 | 185,070 |
|  | MM "Mealtimes" | 817 |
|  | TX "food assistance programs" OR "meal preparation" | 395 |
|  | TX (“Malnutrition” OR “malnourished” OR maln* OR “undernutrition” OR “poor nutrition status” OR “nutrition care” OR “nutrition care pathway” OR “identify malnutrition” OR “detect malnutrition” OR “meal provision” OR “meal preparation” OR grocery OR “grocery shopping” OR “grocery provision” OR “food shopping”) | 7,378 |
|  | TX (“meals on wheels” OR “home-delivered meals” OR “community meals”) | 156 |
|  | S7 OR S8 OR S9 OR S10 | 8,346 |
|  | MM "Elder Care" OR DE "Home Environment" OR MM "Self-Care Skills" | 20,227 |
|  | TI ( “Elderly” OR “older adult” OR senior* OR geriatric* OR “aged individual” OR “aged people” OR “aging” OR “older people” OR “older individual” OR “over 65” OR “sixty five and over” ) OR AB ( “Elderly” OR “older adult” OR senior* OR geriatric* OR “aged individual” OR “aged people” OR “aging” OR “older people” OR “older individual” OR “over 65” OR “sixty five and over” ) | 168,414 |
|  | TI ( “ageing” OR “homebound older” OR “house bound older” OR “frailty” ) OR AB ( “ageing” OR “homebound older” OR “house bound older” OR “frailty” ) | 14,297 |
|  | S12 OR S13 OR S14 | 192,587 |
|  | MM "Qualitative Methods" | 7,004 |
|  | TI ( Qualitative OR “mixed methods” OR “observational study” OR “survey” OR “case studies” OR “evaluation methods” OR interview* OR “focus group*” OR “naturalistic observation” OR “participant observation” OR “social science research” OR transcript* OR ethnography* OR phenomenol* OR “grounded theory*” OR “purposive sample” OR hermeneutic* OR heuristic* OR “lived experience*” OR narrative* OR “life experience*” OR “life stor*” OR “cluster sample” OR “action research” OR “observational method” OR ... | 967,764 |
|  | S16 OR S17 | 968,035 |
|  | S6 AND S11 AND S15 AND S18 | 65 |

Scopus search 18/08/2023

( ( TITLE-ABS-KEY ( "Qualitative research" ) OR TITLE-ABS-KEY ( "Qualitative" OR "mixed methods" OR "observational study" OR "survey" OR "case studies" OR "evaluation methods" OR interview* OR "focus group*" OR "naturalistic observation" OR "participant observation" OR "social science research" OR transcript* OR ethnography* OR phenomenol* OR "grounded theory*" OR "purposive sample" OR hermeneutic* OR heuristic* OR "lived experience*" OR narrative* OR "life experience*" OR "life stor*" OR "cluster sample" OR "action research" OR "observational method" OR "content analysis" OR "thematic analysis" OR "narrative analysis" OR "constant comparative method" OR "field stud*" OR "fieldnotes" OR "audio recording" OR "video recording" OR "theoretical sample" OR "discourse analysis" ) ) ) AND ( ( TITLE-ABS-KEY ( "Frail elderly" OR "aged" ) OR TITLE-ABS-KEY ( "Elderly" OR "older adult" OR senior* OR geriatric* OR "aged individual" OR "aged people" OR "aging" OR "older people" OR "older individual" OR "over 65" OR "sixty five and over" ) OR TITLE-ABS-KEY ( "ageing" OR "homebound older" OR "house bound older" OR "frailty" ) ) ) AND ( ( TITLE-ABS-KEY ( "Food Assistance" OR "Meals-on-Wheels" OR "Meal Preparation" OR "Meals" ) OR TITLE-ABS-KEY ( "Malnutrition" OR "malnourished" OR maln* OR "undernutrition" OR "poor nutrition status" OR "nutrition care" OR "nutrition care pathway" OR "identify malnutrition" OR "detect malnutrition" OR "meal provision" OR "meal preparation" OR grocery OR "grocery shopping" OR "grocery provision" OR "food shopping" ) OR TITLE-ABS-KEY ( "meals on wheels" OR "home-delivered meals" OR "community meals" ) ) ) AND ( ( TITLE-ABS-KEY ( "Volunteer Workers" OR "Volunteer Experiences" OR "Communities" OR "Community Networks" OR "Home Health Aides" ) OR TITLE-ABS-KEY ( "Community volunteers" OR "community volunt*" OR "volunteers" OR "voluntary provider" OR "community voluntary provider" OR "Non-governmental organi#ation" OR "NGO" ) OR TITLE-ABS-KEY ( "community health" OR "community care" OR "domiciliary care" OR "domiciliary support" ) ) ) AND ( LIMIT-TO ( DOCTYPE , "ar" ) )

Pubmed search 18/08/2023

| 16 | #4 AND #8 AND #12 AND #15 | 182 |
| --- | --- | --- |
| 15 | #13 OR #14 | 2,627,425 |
| 14 | Qualitative[Title/Abstract] OR "mixed methods"[Title/Abstract] OR "observational study"[Title/Abstract] OR "survey"[Title/Abstract] OR "case studies"[Title/Abstract] OR "evaluation methods"[Title/Abstract] OR interview*[Title/Abstract] OR "focus group*"[Title/Abstract] OR "naturalistic observation"[Title/Abstract] OR "participant observation"[Title/Abstract] OR "social science research"[Title/Abstract] OR transcript*[Title/Abstract] OR ethnography*[Title/Abstract] OR phenomenol*[Title/Abstract] OR "grounded theory*"[Title/Abstract] OR "purposive sample"[Title/Abstract] OR hermeneutic*[Title/Abstract] OR heuristic*[Title/Abstract] OR "lived experience*"[Title/Abstract] OR narrative*[Title/Abstract] OR "life experience*"[Title/Abstract] OR "life stor*"[Title/Abstract] OR "cluster sample"[Title/Abstract] OR "action research"[Title/Abstract] OR "observational method"[Title/Abstract] OR "content analysis"[Title/Abstract] OR "thematic analysis"[Title/Abstract] OR "narrative analysis"[Title/Abstract] OR "constant comparative method"[Title/Abstract] OR "field stud*"[Title/Abstract] OR "fieldnotes"[Title/Abstract] OR "audio recording"[Title/Abstract] OR "video recording"[Title/Abstract] OR "theoretical sample"[Title/Abstract] OR "discourse analysis"[Title/Abstract] | 2,622,424 |
| 13 | "qualitative research"[MeSH Terms] | 82,873 |
| 12 | #9 OR #10 OR #11 | 709,506 |
| 11 | "ageing"[Title/Abstract] OR "homebound older"[Title/Abstract] OR "house bound older"[Title/Abstract] OR "frailty"[Title/Abstract] | 80,275 |
| 10 | "Elderly"[Title/Abstract] OR "older adult"[Title/Abstract] OR senior*[Title/Abstract] OR geriatric*[Title/Abstract] OR "aged individual"[Title/Abstract] OR "aged people"[Title/Abstract] OR "aging"[Title/Abstract] OR "older people"[Title/Abstract] OR "older individual"[Title/Abstract] OR "over 65"[Title/Abstract] OR "sixty five and over"[Title/Abstract] | 654,880 |
| 9 | (elderly, frail[MeSH Terms]) OR (adult, frail older[MeSH Terms]) | 15,126 |
| 8 | #5 OR #6 OR #7 | 94,962 |
| 7 | ("meals on wheels"[Text Word] OR "home-delivered meals"[Text Word] OR "community meals"[Text Word]) OR ("food assistance"[Text Word] OR "meal preparation"[Text Word]) | 3,231 |
| 6 | "Malnutrition"[Text Word] OR "malnourished"[Text Word] OR maln*[Text Word] OR "undernutrition"[Text Word] OR "poor nutrition status"[Text Word] OR "nutrition care"[Text Word] OR "nutrition care pathway"[Text Word] OR "identify malnutrition"[Text Word] OR "detect malnutrition"[Text Word] OR "meal provision"[Text Word] OR "meal preparation"[Text Word] OR grocery[Text Word] OR "grocery shopping"[Text Word] OR "grocery provision"[Text Word] OR "food shopping"[Text Word] | 75,814 |
| 5 | (meals on wheels[MeSH Terms]) OR (ready to eat meals[MeSH Terms]) | 18,072 |
| 4 | #1 OR #2 OR #3 | 336,154 |
| 3 | ("community health"[Title/Abstract] OR "community care"[Title/Abstract] OR "domiciliary care"[Title/Abstract] OR "domiciliary support"[Title/Abstract]) OR ("volunteer experience"[Title/Abstract]) | 38,485 |
| 2 | "Community volunteers"[Title/Abstract] OR community volunt*[Title/Abstract] OR volunteers[Title/Abstract] OR "voluntary provider"[Title/Abstract] OR "community voluntary provider"[Title/Abstract] OR "Non-governmental organization"[Title/Abstract] OR "NGO"[Title/Abstract] | 190,300 |
| 1 | (((volunteer workers[MeSH Terms]) OR (communities[MeSH Terms])) OR (community networks[MeSH Terms])) OR (aides, home health[MeSH Terms]) | 123,602 |
