# Supplementary material for: Experiences and perceptions of meals on wheels volunteers in providing nutritional care to older adults: A qualitative evidence synthesis
Source: PLoS One. 2025 Apr 9;20(4):e0315443. doi: 10.1371/journal.pone.0315443 (PMC11981223; doi:10.1371/journal.pone.0315443)
Supplement: Attachment — Submitted filename: Response to Reviewers no. 2- 2025.docx [file pone.0315443.s007.docx]

Dear Editorial team and Reviewers

Thank you for taking the time to review and advise on our manuscript. Please find below a point-by-point response. We hope that you will agree that the article is sufficiently enhanced by addressing each of the valid points raised. We have tracked these changes in the original manuscript and also attach a clean revised manuscript.

| **Additional Editor Comments** | |
| --- | --- |
| *Comments* | *Response* |
| The obvious concern is the small sample - just three primary qualitative studies. However, as you have conducted a thorough search of the literature, the paucity of papers reflects the extant literature and cannot be easily changed, other than by recommending that more such studies are conducted. | We agree and feel that this concern has been addressed in:  Strength and Limitations: lines 463-5  Conclusions: Lines 475-8 |
| Reviewer 5 raises questions of differences between high and low income countries, and urban and rural areas. As the three existing studies are all in high income countries, I do not know if it possible for you to discuss differences between high and low income countries, but please do comment on whether there might be rural/urban differences. | Addressed below. |
| Reviewer 3 mentions the low quality of the figure, but I believe that is an artifact of the process of compiling a reviewer pdf (sorry!) - the actual figure file looks fine to me. | Thank you. |
| Reviewer #3 | |
| The manuscript entitled "Experiences and Perceptions of Meals on wheels volunteers in providing nutritional care to older adults: A qualitative evidence synthesis " is a very informative manuscript and will greatly contribute to the health sector. The manuscript has no major mistakes but the figures are a little blurred and need high resolution. | Thank you. Please refer to the note form the editor. |
| Reviewer #4 | |
| Your manuscript is technically sound, and the data supports the conclusions. The statistical analysis has been performed rigorously and appropriately. All data underlying the findings in your manuscript is fully available. Additionally, the manuscript is presented in a clear manner and written in standard English. | Thank you. |
| Reviewer #5 |  |
| 1. This work incorporates studies performed in the US, UK, and Ireland. These appear to be high income countries. Therefore, a research of this magnitude does not adequately assess the validity of MoW which can more or less be evident among lower income nations. So the sample size used does not comprehensively underpin the methodology. | Thank you for raising this concern. We have included some more detail in Strengths and Limitations to discuss the paucity of evidence from lower income countries.  Lines 466-472: “Furthermore, the included studies were conducted in high-income countries, and it is uncertain whether the findings would be replicated in lower- and middle-income countries. The authors noted that the articles screened at full text stage were all performed in higher income countries. This indicates a paucity of knowledge on this issue from global areas where the need for Meals on Wheels service may be significantly higher, that is noted in other systematic reviews synthesising the evidence of social and economic factors and malnutrition or the risk of malnutrition in older adults[1].” |
| 2. Little evidence is detailed as to whether the study compares MoW in urban vs. rural districts. If little or no data/findings are available to address these points, this should be at least mentioned. | Thank you for the comment. However, this information has been provided in the Results: Lines 193-202 “O’Dwyer and Timonen, 2009 aimed to examine the motivations of Irish MoW volunteers and the nature of their contributions in order to explore key considerations and prepare for the future role of volunteering within the service across **13 sites (n= 5 rural and n= 8 urban);** Thomas et al, 2021 aimed to describe the interactions of MoW drivers with their clients, and the perceived benefits of the program to the volunteers who make it possible across **six sites in the US (n= 4 rural, n= 5 suburban and n= 3 urban);** and, Papadaki et al 2021 aimed to explore the perceptions of MoW service providers in England, with regard to the benefits of MoW and the challenges faced by the service in **two local authorities (n= 23 urban and n= 20 semi-urban)** located in South-West England.” As such, we feel that there is a balance reflected in rural vs urban districts within the included studies. |
| Reviewer #6 | |
| Thank-you for giving me the opportunity to review this paper. It appears that all the previous reviewers' comments have been addressed as substantial revision to the paper has been made. The literature search process is thorough, and the findings explored.  The paper reports a review of three reviews with some analysis of the publications reviewed. The decision as to whether this type of paper fits the scope of the journal lies with the editors. | Thank you. |
