# Supplementary material for: Experiences and perceptions of meals on wheels volunteers in providing nutritional care to older adults: A qualitative evidence synthesis
Source: PLoS One. 2025 Apr 9;20(4):e0315443. doi: 10.1371/journal.pone.0315443 (PMC11981223; doi:10.1371/journal.pone.0315443)
Supplement: Attachment — Submitted filename: Response to Reviewers.docx [file pone.0315443.s006.docx]

Dear Editorial team and Reviewers

Thank you for taking the time to review and advise on our manuscript. Please find below a point-by-point response. We hope that you will agree that the article is sufficiently enhanced by addressing each of the valid points raised. We have tracked these changes in the original manuscript and also attach a clean revised manuscript.

| **Journal Requirements** | |
| --- | --- |
| *Requirements* | *Response* |
| 1. Style requirements | The manuscript has been amended to comply with the style requirements. A separate title page is now included. Figures are not included in-text. |
| 2. Supporting Information files | Captions for Supporting Information files inserted at the end of manuscript, and updated in-text citations to match accordingly. |
| 3. PRISMA Checklist | Please refer to previous correspondence with Irene Nathalie Fernandez Tolentino <irene.tolentino@straive.com> , July 31, 2024 (Appendix 1).  It was agreed that as the submitted article is a qualitative evidence synthesis, deriving data from observation, interviews, and focused on meanings and interpretations of participants.  1. The reporting of the article is therefore supported by the ENTREQ checklist enhancing transparency in reporting the synthesis of qualitative research (<https://bmcmedresmethodol.biomedcentral.com/articles/10.1186/1471-2288-12-181#citeas>, submitted as a supplemental file). Do you still require an additional checklist of the PRISMA.  2. 'Qualitative Evidence Synthesis' is in the article title, that clearly identifies the type of review provided.  3. A PRISMA flowchart is figure 1 as indicated in the uploaded manuscript. |
| 4. Reference list | This is up to date. |
| **Additional Editor Comments** | |
| *Comments* | *Response* |
| 1. Interpretation of Volunteer Burden | A sentence has been added to the discussion to acknowledge the wealth of existing evidence that home-delivered meals have directly on the nutritional intake of older adults in the previous 5 years and support the positive impact of volunteers in supporting nutrition care. Some rewording has also been suggested to temper against underestimation of this impact, for example “eclipsed” rather than “overshadowed” to describe theme 2; removing words with conflict connotations (e.g. combat, struggle, etc.). |
| 2. Scope of Impact on Nutrition Care | Text and supporting references in the introduction have been redacted where the statements were describing disease-related malnutrition and dietary interventions that are more clinically orientated and beyond the scope of a lay person volunteer role.  A re-examination of the codes (added as S2-Table 2) finds that there are two codes directly (relating to meals) and four codes that indirectly (relating to psychosocial and welfare parameters of nutrition care) related to the provision of nutrition care.  The aim of the included three articles are stated in the section “Descriptive characteristics of included studies” and we address the need to directly report perspectives among the volunteers provision of care in the introduction.  The introduction acknowledges the role of community volunteers of mitigating the risk of food insecurity among older adults. |
| 3. Generalization of Challenges Across All MoW Programs | Thank you for this observation. Wording has been amended as advised throughout the discussion to ensure that the argument is couched within the confines of limited data. |
| 4. Limited Data Leading to Broad Conclusions | As above with point 3. |
| 5. Thematic Synthesis and Subjectivity | A reflexive statement has been added to expand on the previous limited description of how we aimed to be objective in identifying themes. |
| 6. Enhancing Methodological Rigor | More detail has been added to the section “data synthesis”. The methods section has been rewritten in parts for better clarity and description of the QES approach (see tracked changes). The codebook has been added as S2-table. Finally, the ENTREQ reporting checklist has been doublechecked (S1-table). |
| 7. Discussion and Conclusion |  |
| a. Practical Implications: | A paragraph suggesting enhanced training, increased awareness and advocacy to better support MoW services has been added to the discussion. |
| b. Recommendations for Future Research | Recommendation for further research including suggested study designs have been added to the discussion. |
| 8. Minor Revisions |  |
| a. Consistency and Clarity | Thank you, consistency in use of abbreviations (e.g. MOW) and terms (e.g. food insecurity/security) has been double-checked. |
| b. Grammar and Syntax | The manuscript has been revised and we hope that it reads more fluently now. |
| Reviewer #1 | |
| Why focus only on qualitative? | A sentence has been added to the discussion to acknowledge the wealth of existing evidence that home-delivered meals have directly on the nutritional intake of older adults in the previous 5 years and support the positive impact of volunteers in supporting nutrition care. We address the need to directly report perspectives and experiences among the volunteers provision of care, in the introduction. |
| Reviewer #2 | |
| Your idea for research is both novel and insightful, and it has given me fresh insights into the topic. | Thank you. |

Appendix 1: Previous correspondence with editorial team regarding use of ENTREQ
